# Sugar Metabolism of *Scardovia wiggsiae*, a Novel Caries-Associated Bacterium

**DOI:** 10.3389/fmicb.2020.00479

**Published:** 2020-03-25

**Authors:** Mai Kameda, Yuki Abiko, Jumpei Washio, Anne C. R. Tanner, Christine A. Kressirer, Itaru Mizoguchi, Nobuhiro Takahashi

**Affiliations:** ^1^Division of Orthodontics and Dentofacial Orthopaedics, Tohoku University Graduate School of Dentistry, Sendai, Japan; ^2^Division of Oral Ecology and Biochemistry, Tohoku University Graduate School of Dentistry, Sendai, Japan; ^3^Forsyth Institute, Cambridge, MA, United States; ^4^Harvard School of Dental Medicine, Boston, MA, United States

**Keywords:** *Scardovia*, acid production, fluoride, Bifid shunt, metabolome, caries, dentistry

## Abstract

*Scardovia wiggsiae* has been detected from caries in children and adolescents and has been suggested to be a caries-associated microorganism. To investigate the cariogenic potential of *S. wiggsiae*, we examined carbohydrate metabolism and acid productivity, the fluoride sensitivity of carbohydrate metabolism and the mechanism by which fluoride inhibits carbohydrate metabolism, and the acid sensitivity of carbohydrate metabolism in this bacterium. *S. wiggsiae* metabolized glucose and reduced the environmental pH to 3.5. It mainly produced acetic acid from glucose, together with small amounts of lactic and formic acid. The 50% inhibitory concentration of fluoride for acid production was 8.0 mM at pH 7.0 and 1.5 mM at pH 5.5, which were much higher than those of representative caries-associated bacteria, such as *Streptococcus mutans*. Metabolomic profiles showed the accumulation of 3-phosphoglycerate and a marked reduction in the pyruvate concentration in the presence of fluoride, suggesting that fluoride inhibits the latter half of glycolysis, including enolase activity. Enolase activity was inhibited by fluoride in *S. wiggsiae*, but it was more fluoride-tolerant than the enolase activity of *S. mutans*. Unlike in *S. mutans*, lactic acid did not inhibit acid production by *S. wiggsiae* at acidic pH. These results indicate that *S. wiggsiae* exhibits high acid production and tolerance to fluoride and lactic acid. *S. wiggsiae* possesses a unique metabolic pathway, the F6PPK shunt, which might allow it to avoid the lactate-formate pathway, including fluoride-sensitive enolase activity, and enable metabolic flow to the fluoride-tolerant acetate pathway. The fluoride tolerance of *S. wiggsiae’s* enolase activity also increases the fluoride tolerance of its carbohydrate metabolism. The lactic acid tolerance of *S. wiggsiae’s* acid production might result in *S. wiggsiae* having high acidogenic and aciduric potential and make it ecologically competitive in acidic environments, such as caries lesions, where lactic acid predominates.

## Introduction

Many children in low income families suffer from early childhood caries (ECC), which remains a public health problem worldwide (Kasebaum et al., 2015). Such lesions progress rapidly and destroy the primary dentition, and moreover, they increase the risk of caries in the permanent teeth. Furthermore successful treatment is difficult and as many as 50% of children relapse after treatment (Tanner et al., 2011; Hajishengallis et al., 2017). The oral biofilm is involved in the formation of ECC and is derived from various factors, such as oral bacteria on the dentin surface, saliva, and dietary carbohydrates (Hajishengallis et al., 2017). *Scardovia wiggsiae* in addition to *Streptococcus mutans* and other acid-producing bacteria, have also been associated with white spot initial carious lesions and aggressive caries in adolescents (Tanner et al., 2012; Eriksson et al., 2018).

While *S. wiggsiae* has been detected in ECC without *S. mutans*, a bacterial complex including *S. mutans* and *S. wiggsiae* characterized adolescents with active caries (Eriksson et al., 2018). *S. wiggsiae* was isolated from root caries in adults expanding the age range of patients with *S. wiggsiae* associated carious lesions (Mantzourani et al., 2009). Metabolically *S. wiggsiae* contributes to the acidification of the oral biofilm and is receiving increasing attention as a caries-associated bacterium (Hajishengallis et al., 2017). Although it is recognized that caries-associated bacteria such as *S. mutans* and *lactobacilli* can cause caries, it has been reported that other caries-associated bacteria, including *S. wiggsiae*, can cause caries even in the absence of the former bacteria, suggesting that it is necessary to clarify the cariogenic potential of the latter bacteria (Chandna et al., 2018).

*Scardovia* is one of the 7 genera of the *Bifidobacteriaceae* family. It is a new bacterial genus and was separated from the genus *Bifidobacterium* in 2002 due to differences in its genome sequence (Jian and Dong, 2002). *S. wiggsiae* was detected in severe ECC as clone CX010 in 2002 (Becker et al., 2002). Despite limited information about the metabolic pathways of *S. wiggsiae*, the species mainly produces acetate from glucose. This suggests that, like oral *Bifidobacterium* species, *S. wiggsiae* possesses a unique metabolic pathway called the fructose-6-phosphate pathway (F6PPK shunt) (Ruas-Madiedo et al., 2005; Sánchez et al., 2005; Manome et al., 2019), which differs from the glycolytic pathway the caries-associated *S. mutans*. However, the biochemical properties of carbohydrate metabolism in *S. wiggsiae* remains incomplete. Thus, the purpose of this study was to examine *S. wiggsiae* for (1) carbohydrate metabolism activity and acid productivity, (2) the fluoride sensitivity of carbohydrate metabolism and the mechanism through which fluoride inhibits carbohydrate metabolism, and (3) the acid sensitivity of carbohydrate metabolism.

## Materials and Methods

### Bacterial Strains and Growth Conditions

*S. wiggsiae* C1A55^T^ and *S. mutans* NCTC10449^T^ were used. These bacteria were maintained on CDC anaerobe blood agar (Nippon BD, Tokyo, Japan) at 37°C in an anaerobic glove box (N_2_, 80%; H_2_, 10%; CO_2_, 10%; NHC-type; Hirasawa Works, Tokyo, Japan). *S. wiggsiae* C1A55 was cultured in a brain heart infusion (Becton Dickinson, Franklin Lakes, NJ, United States), supplemented with 0.5% glucose and 2% lamb serum, at 37°C in the NHC-type box, while *S. mutans* NCTC10449 was cultured in a tryptone-yeast extract-glucose medium, containing 50 mM potassium phosphate buffer (PPB, pH 7.0) and 0.5% glucose. The bacteria were harvested by centrifugation (15,000 × g, 10 min, 4°C) during the logarithmic growth phase, using double-sealed centrifuge tubes to maintain anaerobic conditions. Then, the bacterial cells were washed with 2 mM PPB (pH 7.0), containing 150 mM KCl and 5 mM MgCl_2_, resuspended in the same buffer at an optical density (660 nm) of 3.5 and stored at 4°C prior to use. All of the subsequent procedures, including the washing and preservation of the cells, were carried out in an anaerobic glove box (N_2_, 90%; H_2_, 10%; NH-type; Hirasawa Works).

### Bacteria-Induced pH Reduction and Its Sensitivity to Fluoride

A pH-stat system (AUTO pH-stat, model AUT-211S; Toa Electronics, Tokyo, Japan) was used to measure the pH of the reaction mixture, which contained 2.8 mL of the cell suspension. The pH of the mixture was adjusted to 7.0 by adding 60 mM KOH, as described previously (Kawashima et al., 2013), before the mixture was pre-incubated at 37°C for 3 min. Potassium fluoride (KF) was added to the mixture at a final concentration of 0–25 mM (1 mM = 19.1 ppmF), and then the mixture was further pre-incubated for 4 min. The reduction in pH was started by adding 10 mM glucose, and the pH was measured for 50 min.

### Bacterial Acid Production and Its Sensitivity to Fluoride

The reaction mixture containing 2.8 mL of the cell suspension was set to a pH-stat (AUTO pH-stat; model AUT-211S, Toa Electronics) and was adjusted to 7.0 or 5.5 by adding 0.12 N HCl, as described previously (Kawashima et al., 2013), before the reaction mixture was pre-incubated at 37°C for 3 min. KF was added to the reaction mixture at a final concentration of 0–20 mM, before the mixture was subjected to further pre-incubation for 4 min. Acid production was started by adding glucose at a final concentration of 10 mM. The rate of acid production was monitored for 10 min, based on a titration volume of 60 mM KOH, using the pH-stat system. The 50% inhibitory concentration (IC_50_) of fluoride was also calculated.

### Acidic End-Products

The concentrations of acidic end-products were analyzed, as described previously (Takahashi et al., 1987; Norimatsu et al., 2015). In the experiment described above, the reaction mixture (0.45 mL) was sampled before and 10 min after the addition of glucose. The samples were immediately mixed with 0.05 mL of 6N perchloric acid to terminate bacterial metabolism, before being removed from the anaerobic box and filtered through a polypropylene membrane (pore size: 0.20 μm; Toyo Roshi Ltd., Tokyo, Japan). The filtrates were quantitatively analyzed using high-performance liquid chromatography (Shimadzu Prominence LC-20AD, Shimadzu Co., Ltd., Kyoto, Japan).

### Metabolome Analysis

The bacterial suspension was incubated with glucose for 2 min, as described above, in the presence or absence of 5 mM (pH 7.0) or 2 mM (pH 5.5) KF. Before and after the incubation procedure, the reaction mixture was sampled and immediately centrifuged at 10,000 rpm for 2 min in order to separate the cell fraction and supernatant. The metabolites in the cells were extracted from the cell fraction and pre-treated, as reported previously (Takahashi et al., 2010). The extracted metabolites were analyzed using capillary electrophoresis and time-of-flight mass spectrometry (CE-TOFMS; G1600AX and G1969A; Agilent Technologies, Waldbronn, Germany), as described previously (Takahashi et al., 2010). The obtained metabolomic data were analyzed using specific software (MassHunter workstation; Agilent Technologies, CA, United States) and 35 metabolites were identified as described previously. The following metabolites were particularly targeted: glucose 6-phosphate (G6P), fructose 6-phosphate (F6P), 3-phosphoglycerate (3PG), phosphoenolpyruvate (PEP), and pyruvate, which are involved in glycolysis, and erythrose 4-phosphate (E4P), sedoheptulose 7-phosphate (S7P), ribose 5-phosphate (Ribo5P), and ribulose 5-phosphate (Ribu5P), which are involved in the F6PPK shunt.

### Inhibition of Enzyme Activity by Fluoride

*S. wiggsiae* cells were harvested by centrifugation during the logarithmic growth phase, washed twice with 2 mM PPB (pH 7.0), containing 150 mM KCl and 5 mM MgCl_2_, and stored at −80°C as a pellet prior to use. The cells were suspended in 2 mM PPB, containing 1 mM dithiothreitol, and then disrupted by sonic oscillation for 10 min at 4°C (200 W, 2 A; Insonator, Kubota, Japan). Cell debris was removed by centrifugation (10,000 × g, 10 min, 4°C), and the enzymatic activity of the resultant cell-free extract (CFE) was assayed. The assay mixture contained 20 mM 2-phosphoglycerate; the CFE; and 0, 2, 5, or 20 mM KF (pH 7.0) in 2 mM PPB (pH 7.0) (Guha-Chowdhury et al., 1997; Takahashi et al., 1997). Enzymatic activity was measured by monitoring the production of PEP from 2-phosphoglycerate at 240 nm using a spectrometer (UV-1800; Shimadzu Co., Ltd.).

### Effects of Acid on Bacterial Acid Production

The reaction mixtures containing 2.8 mL of cell suspension was set to a pH-stat system and adjusted to pH 7.0 or 5.5. Acetate buffer or lactate buffer was added to the reaction mixture at a final concentration of 0–100 mM, and the pH of the reaction mixture was adjusted to 7.0 or 5.5 by adding HCl or KOH, before the reaction mixture was pre-incubated at 37°C for 7 min. Then, 10 mM glucose was added to the mixture, and acid production was monitored as described above.

### Statistical Analyses

The data are expressed as mean and standard deviation values and were analyzed using the paired *t*-test, the paired *t*-test combined with Bonferroni’s correction, or Dunn’s test. Differences associated with *P* < 0.05 were considered to be statistically significant.

## Results

### Bacterial Acid Production and Its Tolerance to Fluoride

**FIGURE 1:**
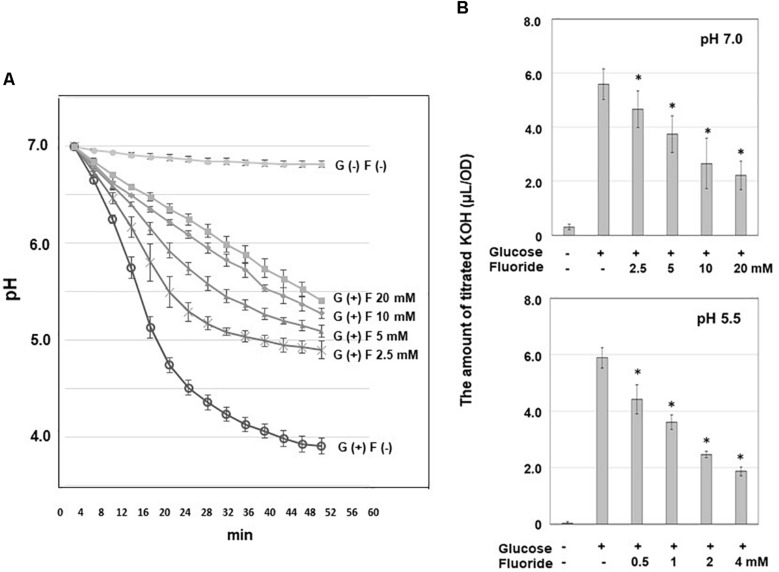
Acid production from glucose by *S. wiggsiae* and inhibitory effects of fluoride on such acid production. **(A)** Curves of the reduction in pH from pH 7.0; **(B)** the rates of acid production at pH 7.0 and 5.5. The data are shown as the mean and standard deviation of three independent experiments. Fifty percent inhibitory concentrations (IC_50_) were calculated from the fluoride concentration and the rate of acid production. The significance of difference from the control was analyzed using Dunn’s test (*P < 0.05).

Since the acidification by bacterial sugar metabolism on the tooth surface is a causative factor of dental caries, the pH reduction after the addition of sugar to bacterial suspension was first analyzed in the present study. The pH remained above pH 6.5 in the absence of glucose, but after the addition of glucose the pH immediately decreased and fell below the critical pH of enamel (pH 5.5), reaching pH 3.56 within 2 h (Figure 1A). The addition of KF inhibited the reduction in pH in a concentration-dependent manner.

The acid production produced from glucose by *S. wiggisae* was monitored based on titration volume of alkali (KOH) solution which is equivalent to the amount of acid production by using a pH-stat (Figure 1B). The acid production rate of *S. wiggsiae* was 5.9 ± 4.0 μL/OD at pH 5.5 and 5.6 ± 0.6 μL/OD at pH 7.0, i.e., *S. wiggsiae* exhibited similarly high acid production rates at pH 7.0 and 5.5 (Figure 1B). The addition of KF inhibited acid production in a concentration-dependent manner, but at pH 7.0 acid production continued even at high KF concentrations (20 mM) (Figure 1B). Based on these findings, the IC_50_ of fluoride for acid production was calculated, and it was found to be 8.0 mM at pH 7.0 and 1.5 mM at pH 5.5.

### Acidic End-Products

Since the profile of acidic end-products suggests metabolic pathways and inhibition steps by KF, acidic end-products were analyzed. Acetic acid accounted for about 70% of the acidic end-products produced from glucose, with the remaining acidic end-products being formic acid at pH 7.0 and formic acid and lactic acid at pH 5.5 (Table 1). The addition of KF resulted in a significant decrease in the level of acidic end-products, which was accompanied by an increase in the percentage of acid end-products accounted for by acetic acid to 85–90% and reductions in the percentages of acid end-products accounted for by formic and lactic acid.

**TABLE 1:** Metabolic end-products from glucose at pH 7.0 and 5.5 by *S. wiggsiae*.

Acidic end-products				
	Substrate	Acetate	Formate	Lactate
pH 7.0	G (−) F (−)	27.2 ± 5.5^†^ (78.4)^‡^	7.52 ± 1.8 (21.6)	ND
	G (+) F (−)	90.4 ± 8.9 (74.4)	31.1 ± 3.5 (25.6)	ND
	G (+) F (2.5)	80.3 ± 8.1 (74.4)**	27.6 ± 3.7 (25.6)	ND
	G (+) F (5)	67.1 ± 8.3 (76.0)**	21.1 ± 2.9 (24.0)**	ND
	G (+) F (10)	55.8 ± 11.7 (81.6)**	12.6 ± 8.2 (18.4)**	ND
	G (+) F (20)	54.0 ± 7.4 (85.1)**	9.46 ± 2.9 (14.9)**	ND
pH 5.5	G (−) F (−)	27.6 ± 5.8 (78.5)	7.56 ± 2.1 (21.5)	ND
	G (+) F (−)	104.8 ± 11.1 (71.4)	22.9 ± 3.1 (15.6)	19.0 ± 1.8 (12.9)
	G (+) F (0.5)	86.9 ± 9.2 (74.7)**	25.5 ± 2.6 (21.9)*	3.97 ± 0.9 (3.4)*
	G (+) F (1)	76.0 ± 7.6 (77.7)**	21.8 ± 2.5 (22.3)	ND
	G (+) F (2)	63.0 ± 7.9 (82.3)**	13.6 ± 2.9 (17.7)**	ND
	G (+) F (4)	67.4 ± 9.6 (91.2)**	6.54 ± 1.1 (8.8)**	ND

^†^Data are presented as the mean and standard deviation of three independent experiments (μM/OD). ^‡^Relative amount of each acidic end-product compared with the total amount of acidic end-products (%). G, 10 mM glucose; F, fluoride (mM). *P < 0.05; **P < 0.01; compared with the amount of acidic end-products produced in the presence of glucose.

### Effects of Fluoride on the Metabolomic Profile

**FIGURE 2:**
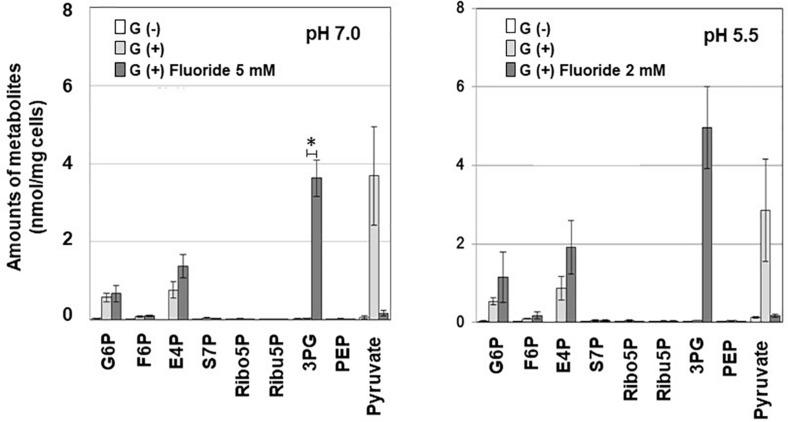
Effects of fluoride on the metabolomic profile of *S. wiggsiae* at pH 7.0 and 5.5. Data are shown as the mean and standard deviation of three independent experiments. The significance of differences between the with and without fluoride conditions were analyzed using the paired *t*-test. The level of significance was set at 0.00556, based on Bonferroni’s correction (*P < 0.0056).

To investigate the mechanism of inhibition of carbohydrate metabolism, metabolomic analysis was performed on G6P and F6P in the process of glucose uptake and phosphorylation, E4P, S7P, Ribo5P, Ribu5P, and 3PG in the F6PPK shunt, and PEP and Pyruvate in the latter half of the glycolytic pathway. When fluoride was added during glucose metabolism, 3PG accumulated, and the level of pyruvate decreased (Figure 2). Similar trends were observed at pH 7.0 and pH 5.5, and significant accumulation of 3PG was seen after the addition of fluoride at pH 7.0. The accumulation of G6P and E4P was also observed after the addition of fluoride, but these changes were not statistically significant.

### Inhibition of Enolase Activity by Fluoride

Since the inhibition of enolase activity by fluoride was predicted from the metabolomic profile, enolase activity was assayed in cell extracts, and its activity was examined at pH 7.0 after the addition of KF. The addition of fluoride inhibited enolase activity in a concentration-dependent manner, i.e., fluoride inhibited enolase activity by < 10% at 2 mM (Table 2) and by 50% (IC_50_) at 4.6 mM (data not shown).

**TABLE 2:** Inhibition of enolase activity by fluoride.

Fluoride concentration	Inhibition of enolase activity by fluoride (%)		
(mM)	*S. wiggsiae*	*B. dentium*	*B. dentium*
0.02	nt	10.4 ± 2.5	7.6 ± 3.3
0.2	nt	49.4 ± 4.7	7.6 ± 3.3
2	8.53 ± 4.7	96.2 ± 1.0	94.9 ± 2.5
5	55.5 ± 4.8	nt	nt
20	92.5 ± 1.8	nt	nt

*Data are shown as the mean ± standard deviation of three independent experiments. Data of B. dentium and S. mutans are adopted from Manome et al. (2019). nt, not tested.*

### The Effect of Acid on Bacterial Acid Production

**FIGURE 3:**
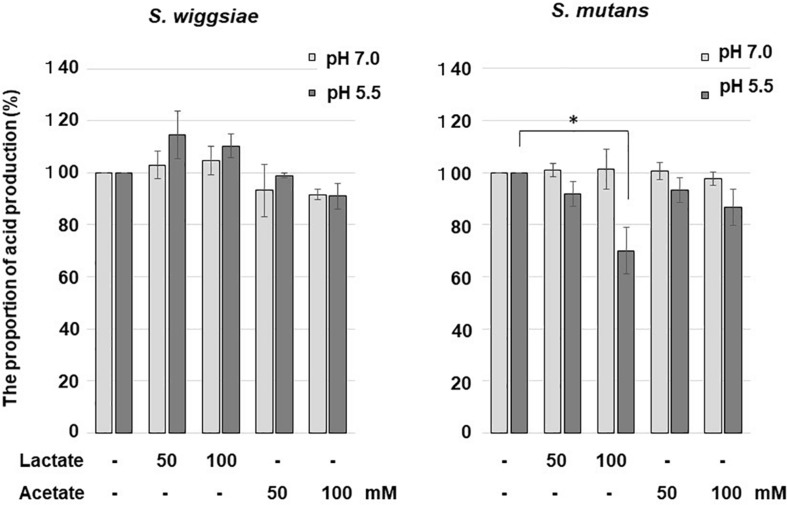
Effects of acetic and lactic acids on acid production from glucose by *S. wiggsiae*. The significance of differences from the control was analyzed using Dunn’s test (*P < 0.05).

At pH 7.0, neither lactic acid nor acetic acid affected carbohydrate metabolism in *S. mutans* or *S. wiggsiae* (Figure 3). At pH 5.5, lactic acid inhibited the acid production of *S. mutans*, whereas acetic acid did not affect acid production in either bacterium.

## Discussion

*S. wiggsiae* produced acid from glucose and reduced the environmental pH to 3.5 (Figure 1A), demonstrating that this bacterium exhibits high acid productivity and tolerance to acidic conditions. In addition, the rate of acid production was similar at both pH 7.0 and 5.5 (Figure 1B), indicating that the acid production pathways of *S. wiggsiae* are acid-tolerant. This result agrees with the findings of a previous study, which demonstrated that *S. wiggsiae* could grow and produce acid at a low pH (Tanner et al., 2018). In addition, *S. wiggsiae* is primarily an anaerobic bacterium whereas *S. mutans* is a facultative anaerobic bacterium, suggesting that *S. wiggsiae* can survive and continue to produce acid under more mature biofilm with low oxygen concentration. These facts suggest that acid product from *S. wiggsiae* could induce hydroxyapatite demineralization and facilitate caries progression by lowering the pH of the oral biofilm. A previous study found that mutans streptococci, which have high cariogenic potential, can reduce the environmental pH to 3.5 (Aizawa et al., 2009), which suggests that the acid productivity and acid tolerance of *S. wiggsiae* are equivalent to those of *S. mutans*. This might be one of the reasons why *S. wiggsiae* is frequently detected in caries lesions (Tanner et al., 2012; Colombo et al., 2017; Chandna et al., 2018).

**FIGURE 4:**
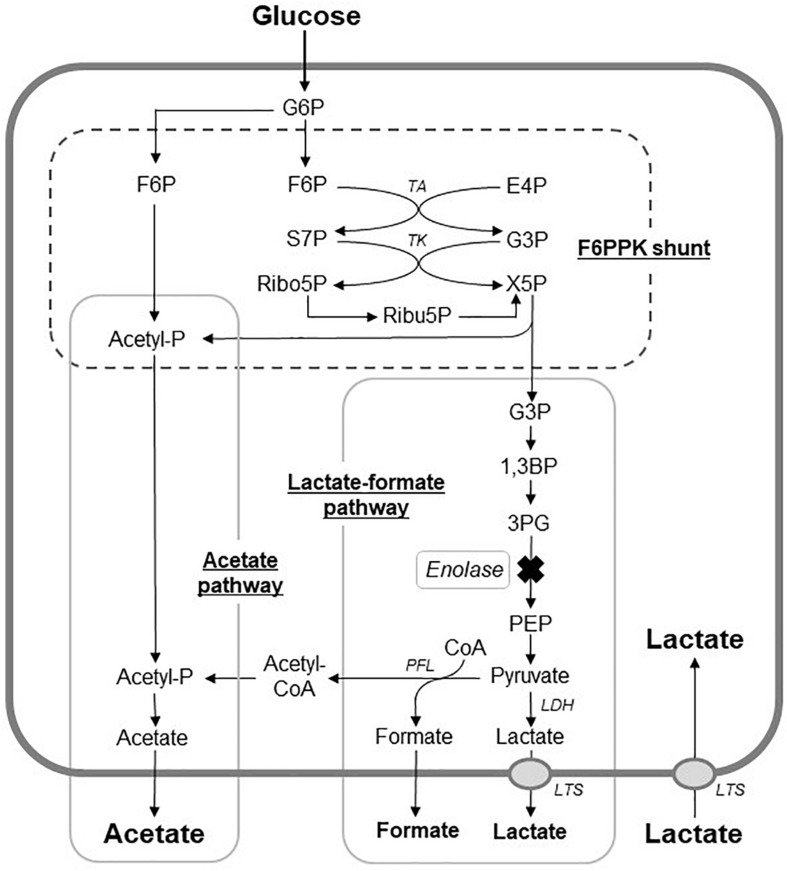
Proposed metabolic pathways for glucose metabolism in *S. wiggsiae*. X mark, a step assumed to be inhibited by fluoride; G6P; glucose 6-phospahte; F6P, fructose 6-phosphate; E4P, erythrose 4-phosphate; S7P, sedoheptulose 7-phosphate; G3P, glyceraldehyde 3-phosphate; X5p, xylulose 5-phosphate; Ribo5P, ribose 5-phosphate; Ribu5P, ribulose 5-phosphate; 1,3BP, 1,3-bisphosphoglycerate; 3PG, 3-phosphoglycerate; PEP, phosphoenolpyruvate; Acetyl-P, CoA, coenzyme A; TA, transalodolase; TK, transketolase; LDH, lactate dehydrogenase; PFL, pyruvate formate-lyase; LTS, lactic acid transport system.

As was reported for *Bifidobacterium dentium* and *Bifidobacterium longum*, acetic acid was the main acidic end-product by *S. wiggsiae* (Table 1), whereas many caries-associated bacteria, such as mutans streptococci, predominantly produce lactic acid (Manome et al., 2019). This suggests that *S. wiggsiae*, like *Bifidobacterium* species, metabolizes sugars via the F6PPK shunt and produces acetic acid as an acidic end-product (Figure 4). In the whole genome sequence of *S. wiggisiae* F0424, the gene sequences of two key enzymes of the F6PPK shunt, transaldolase and transketolase (Figure 4), have been assigned^1^, supporting the involvement of the F6PPK shunt in the sugar metabolism of *S. wiggsiae*. The F6PPK shunt is a metabolic pathway, in which the F6P produced by the phosphorylation of glucose is continuously degraded by the enzymatic activities of transaldolase and transketolase, before it finally enters the latter half of glycolysis via G3P and produces lactic and formic acids (the lactate-formate pathway), or is used to convert acetyl phosphate to acetic acid (the acetate pathway) (Figure 4). In addition to acetic acid, *S. wiggsiae* also produced small amounts of formic and lactic acids (Table 1), indicating that it utilizes both of the abovementioned pathways. Only acetic and formic acids were produced at pH 7.0, while lactic acid was also produced at pH 5.5 (Table 1), suggesting that a metabolic shift between lactic and formic acids occurred in the lactate-formate pathway. Lactate dehydrogenase, responsible for lactic acid production, was reported to function under acidic conditions in oral streptococci, whereas pyruvate formate lyase responsible for formic acid production functions at neutral pH, because of differences between their optimum pH (Iwami et al., 1992). This could also be the case in *S. wiggsiae*. In addition, a previous study found that compared with lactic acid a greater proportion of acetic acid is non-ionized in low pH environments, and hence, acetic acid is more likely to penetrate into enamel and decalcify it from inside than lactic acid (Hoppenbrouwers and Driessens, 1988), suggesting that acetic acid-producing bacteria, such as *S. wiggsiae*, induce caries formation and encourage caries to progress via different mechanisms to lactic acid-producing bacteria, such as *S. mutans*.

The present study revealed that fluoride inhibits acid production by *S. wiggsiae* more efficiently at acidic pH than at neutral pH (Figure 1). Fluoride is present as fluoride ions in the oral cavity, but enters bacterial cells as hydrogen fluoride (Jenkins, 1999). The hydrogen fluoride in bacterial cells changes back to the ionized form of fluoride, which inhibits enolase, an enzyme involved in glycolysis, and hence, carbohydrate metabolism in *Streptococcus*, *Actinomyces*, and *Bifidobacterium* (Hamilton, 1990; Marquis, 1990; Tatevossian, 1990; Jenkins, 1999; Vogel et al., 2002). Since the p*K*a of hydrogen fluoride is 3.17, it is considered that the inhibitory effects of fluoride are increased in acidic environments (Sutton et al., 1987; Jenkins, 1999) because the lower the pH of the surrounding environment the more fluorine ions are converted into hydrogen fluoride, and thus, the easier it is for fluoride to enter bacterial cells. It seems likely that a similar phenomenon occurs in *S. wiggsiae*. The current study demonstrated that the IC_50_ of fluoride for acid production in *S. wiggsiae* was 8.0 mM at pH 7.0 and 1.5 mM at pH 5.5, which are similar to the values obtained for the *Bifidobacterium* strains examined (16.4 mM at pH 7.0 and 1.5 mM at pH 5.5) and much higher than those for *S. mutans* (2.7 mM at pH 7.0 and 0.3 mM at pH 5.5) (Manome et al., 2019), suggesting that carbohydrate metabolism pathways in *S. wiggsiae* are highly tolerant to fluoride.

Fluoride also increased the proportion of acetic acid and decreased the proportions of formic and lactic acids among the acidic end-products (Table 1), indicating that the lactate-formate pathway is more sensitive to fluoride than the acetate pathway. These results are consistent with the metabolomic profile (Figure 2), which showed that fluoride had little effect on the pathway from glucose uptake through G6P to F6P or on the levels of metabolic intermediates in the F6PPK shunt, whereas it did affect the levels of metabolic intermediates in the lactate-formate pathway (Figure 4). The accumulation of 3PG and the apparent reduction in the pyruvate level (Figure 2) suggest that fluoride inhibits enolase, the enzyme that catalyzes the metabolic reaction between 3PG and pyruvate (Figure 4). Indeed, the present study confirmed that fluoride inhibited enolase activity in a concentration-dependent manner; however, 2 mM fluoride inhibited the enolase activity of *S. mutans* and *B. dentium* by 90% (Manome et al., 2019), while it only inhibited the activity of *S. wiggsiae* by <10%, and the IC_50_ value was 4.6 mM, indicating that the enolase of *S. wiggsiae* is more fluoride-tolerant than those of other caries-associated bacteria (Table 2). The fact that *S. wiggsiae* possesses a fluoride-tolerant form of enolase might also make its carbohydrate metabolism activity more tolerant to fluoride. Although the reasons for the fluoride tolerance of *S. weggsiae* remain unclear, it is true that the sensitivity of enolase varies among oral bacteria (Guha-Chowdhury et al., 1997). The mechanism for the difference in fluoride sensitivity might be due to the conformational structure of enolase, since fluoride inhibition is closely related to the binding properties of magnesium, phosphate, and fluoride to the enolase protein (Qin et al., 2006).

In addition to fluoride, acids produced by bacteria can inhibit their carbohydrate metabolism (Carlsson and Hamilton, 1996). In the current study, lactic acid inhibited the acid production of *S. mutans* (Figure 3). This was considered to be due to the lactic acid transport system used by *S. mutans* for lactic acid excretion (Carlsson and Hamilton, 1996; Dashper and Reynolds, 1996) that caused lactic acid to flow into the bacterial cells and acidified the intracellular pH, and hence, inhibited glucose metabolism. In acidic environments some acetic acid becomes undissociated, permeates through the cell membrane, enters bacterial cells and possibly inactivates bacterial metabolism by acidification, but the efficiency of acetic acid penetration is lower than that of the lactic acid transport system, and its inhibitory effects are considered to be negligible (Günther et al., 1991; Carlsson and Hamilton, 1996; Dashper and Reynolds, 1996). In the present study, the acid production of *S. wiggsiae* was not affected by lactic acid (Figure 3), presumably because *S. wiggsiae* does not possess a lactic acid transport system (Günther et al., 1991). In highly cariogenic environments, i.e., acidic environments in which a large amount of lactic acid is present, *S. mutans* might decrease its ability to metabolize carbohydrates and produce acid, whereas *S. wiggsiae* could continue to produce acid, suggesting that *S. wiggsiae* has high acidogenic and aciduric potential. This property might also make *S. wiggsiae* ecologically competitive in acidic environments, such as caries lesions.

## Conclusion

The present study revealed that *S. wiggsiae* has high acid productivity and tolerance to fluoride and lactic/acetic acids. The acid productivity and acid tolerance of *S. wiggsiae* is thought to be equivalent to those of *S. mutans*. *S. wiggsiae* possesses a unique metabolic pathway, F6PPK shunt, which might contribute to its fluoride and acid tolerance by maintaining the metabolic flow to the fluoride-tolerant acetic acid-pathway. The tolerance of acid production to lactic acid may also provide *S. wiggsiae* with high ecological competitiveness in acidic environments such as caries lesions.

## Data Availability Statement

All datasets generated for this study are included in the article/supplementary material.

## Author Contributions

MK contributed to data acquisition and interpretation, drafted, and critically revised the manuscript. YA contributed to conception, design, data acquisition and interpretation, drafted and critically revised the manuscript. JW contributed to conception, design, data interpretation, drafted, and critically revised the manuscript for metabolome analysis CE-TOFMS. AT contributed to conception, design and manuscript review. CK contributed to conception and design. IM contributed to conception and design. NT contributed to conception, design, data interpretation, drafted, and critically revised the manuscript. All authors gave their final approval and agreed to be accountable for all aspects of the work.

## Conflict of Interest

The authors declare that the research was conducted in the absence of any commercial or financial relationships that could be construed as a potential conflict of interest.

## Footnotes

1 https://www.ncbi.nlm.nih.gov/Taxonomy/Browser/wwwtax.cgi?id=857290
